# Transcriptomics Reveals Molecular Features of the Bilateral Pelvic Nerve Injury Rat Model of Detrusor Underactivity

**DOI:** 10.3390/biom13081260

**Published:** 2023-08-18

**Authors:** Jiaxin Wang, Lida Ren, Xinqi Liu, Wenchao Xu, Man Liu, Peng Hu, Tao Wang, Jihong Liu, Qing Ling

**Affiliations:** 1 Department of Urology, Tongji Hospital, Tongji Medical College, Huazhong University of Science and Technology, Wuhan 430030, China; jiaxin@hust.edu.cn (J.W.);; 2 Institute of Urology, Tongji Hospital, Tongji Medical College, Huazhong University of Science and Technology, Wuhan 430030, China; 3 Department of Geriatrics, Tongji Hospital, Tongji Medical College, Huazhong University of Science and Technology, Wuhan 430030, China

**Keywords:** underactive bladder, detrusor underactivity, bilateral pelvic nerve injury, RNA-seq, urodynamics, inflammation, fibrosis

## Abstract

The pathogenesis of detrusor underactivity (DU) is unclear, and the available therapeutic effects are unsatisfactory. We propose to find key molecules and pathways related to DU based on transcriptome sequencing. A rat model of bilateral pelvic nerve injury (BPNI) was established. Bladder tissues from the sham-operated group, 3 and 28 days after BPNI mapping, were taken for urodynamics, histopathology, and RNA-seq. An enrichment analysis of the screened differential expression genes was performed. Three days after BPNI, the results showed urodynamic features of overflow incontinence, while there was a recovery at 28 days after the operation. Masson staining revealed collagen deposition accompanied by progressive thickening of the smooth muscle layer as DU progressed. RNA-seq results suggested that a total of 1808 differentially expressed genes (DEGs) differed among the groups. RNA-seq and subsequent analysis confirmed that the cell cycle and immune response were significantly activated 3 days after BPNI, while extracellular matrix remodeling occurred 28 days after BPNI. Partial DEGs and pathways were verified by qRT-PCR. Validation of key proteins involved in cell cycle, inflammation, and fibrosis was performed by immunohistochemical staining and western blot, respectively. These molecular expression patterns at different time points after BPNI injury provide valuable insights into the search for therapeutic targets for DU.

## 1. Introduction

Detrusor underactivity (DU) is a common lower urinary tract dysfunction that can manifest itself as a dual impairment during the storage and voiding phases [1]. The International Continence Society (ICS) defines DU as a contraction of reduced strength and/or duration, resulting in prolonged bladder emptying and/or failure to achieve complete bladder emptying within a normal time span [2]. Epidemiological studies confirm that 9–48% of men and 12–45% of women undergoing urodynamic evaluation for non-neurogenic lower urinary tract symptoms (LUTS) suffer from DU [3]. Additionally, underactive bladder (UAB) is a general term for a series of symptoms caused by DU, including frequent urination, nocturia, incontinence, weak flow, and intermittency [3,4].

The etiology of DU is very diverse, with iatrogenic DU being a common and intractable complication after pelvic surgery that is mostly caused by potential injury to the nerves innervating the bladder [5]. The nerves that innervate the bladder are close to the rectum and its intrinsic fascia and therefore may cause loss of innervation of the detrusor and decreased bladder sensitivity during proctectomy [6]. Several studies have confirmed the increased absolute incidence of DU after radical prostatectomy, ranging from 13.4% to 42%, and the occurrence of DU may impair functional outcomes and quality of life [7]. Recently, Sun et al. evaluated urodynamics in postoperative patients undergoing radical hysterectomy in non-menopausal women with cervical cancer and found increased residual urine (6.39 ± 10.44 mL vs. 42.32 ± 33.72 mL, *p* < 0.001), decreased bladder compliance (82.63 ± 58.06 mL/cmH_2_O vs. 37.45 ± 28.66 mL/cmH_2_O, *p* < 0.001), and decreased maximum urinary flow rate (25.42 ± 6.46 mL/s vs. 14.43 ± 5.32 mL/s, *p* < 0.001) at 3–6 months postoperatively, as manifested by DU [8]. Although with the widespread use of minimally invasive surgery and the promotion of nerve-preserving procedures, patients are suffering less and less potential injury during surgery, neurogenic DU due to iatrogenic injury is still difficult to completely avoid.

Unfortunately, there is no fundamental treatment for neurogenic DU, and clean intermittent self-catheterization remains the gold standard for reducing high postvoid residual and incomplete voiding [9]. However, intermittent catheterization 4–6 times a day greatly affects the patient’s quality of life and can cause serious complications such as complicated urinary tract infections [4]. Those who cannot tolerate catheterization can only drain the urine through a suprapubic cystostomy. Acetylcholine is the primary neurotransmitter mediating bladder contraction in humans and acts on muscarinic (M3) receptors [10]. Therefore, several parasympathomimetic drugs (e.g., carbachol and bethanechol) have been investigated for the treatment of UAB/DU. However, the ability of these drugs to increase the contractility of the detrusor muscle is extremely limited and is accompanied by side effects that are difficult for patients to tolerate [10,11]. Therefore, the search for pathophysiological changes in bladder tissue during the development of neurogenic DU is clinically important for the development of new DU therapeutic targets.

The bilateral pelvic nerve crush injury (BPNI) model has been widely used in previous studies for the treatment of neurogenic DU [12,13,14]. This model induces loss of innervation of the bladder by clamping the pelvic nerves bilaterally in rats, thus simulating the nerve injury that potentially occurs during pelvic surgery. Ge et al. demonstrated elevated postvoid residual and reduced bladder volume after BPNI, accompanied by increased collagen and reduced smooth muscle, which may be associated with pathway activation of the TGF-β1/Smad pathway [15]. Another study confirmed that BPNI resulted in increased bladder weight, impaired contractility, and reduced smooth muscle content and autonomic innervation [14]. However, previous studies have mostly been limited to urodynamics, pathological changes, and detrusor responsiveness. The pathophysiological mechanisms of neurogenic DU and the overall description of the differences in molecular expression are still less explored.

In this study, we constructed a BPNI rat model, performed urodynamic tests and morphological observations at different postoperative time points, and collected bladder tissue from rats for RNA-seq and subsequent validation, with a view to a comprehensive pathophysiological characterization of neurogenic DU.

## 2. Materials and Methods

### 2.1. Animals and Study Design

All experiments were approved by the Animal Ethics Committee of Tongji Hospital, Tongji Medical College, Huazhong University of Science and Technology (Approval No. TJH-202207004). A total of 45 rats 10–12 weeks old were used for this study and were housed in a standard specific pathogen-free (SPF) class experimental animal facility. The rats were housed at a temperature of 22 ± 2 °C and a relative humidity of 45–55% with a 12-h light and 12-h dark cycle and supplied with food and water ad libitum. After 1 week of acclimatization, the rats were randomly divided into three groups (*n* = 15 per group) and subjected to sham surgery (one group) and BPNI (two groups). A total of 6 rats in each group were selected for urodynamic analysis of their urinary patterns. The bladder tissues of 6 rats in each group were also taken for morphological observation. The rest of the tissues were then collected and frozen at −80 °C for subsequent molecular biology experiments. Finally, bladder tissues from three rats in each group were selected for RNA-seq.

The rats were randomly divided into 3 experimental groups according to the type of surgery and the interval between postoperative evaluations: sham-operated, 3-day post-BPNI, and 28-day post-BPNI. First, we applied awake urodynamics to assess the urinary patterns of rats in each group. Secondly, the morphological characteristics of the rats in each group were compared. Third, the bladders were collected at the respective time points for RNA-seq and bioinformatics analysis, and finally, the bladder tissues of rats in each group were selected for molecular and histological validation.

### 2.2. BPNI Model

Rat BPNI model establishment was performed according to the protocol reported by Dewulf et al. [16]. Briefly, rats were anesthetized using 1.5% isoflurane. The rats were placed in the supine position, and the abdomen was prepared and disinfected by applying iodophor, followed by a midline laparotomy. The prostate was found below the bladder, and the major pelvic ganglion (MPG) and pelvic nerve were located posterolateral to the prostate. The pelvic nerve was separated and crushed 1 cm lateral of the MPG with Dumont #5 forceps for 3 × 15 s (Fine Science Tools, Foster City, CA, USA). Meanwhile, in sham-operated rats, the pelvic nerve was exposed but not crushed. The abdomen was sutured. The bladder was emptied twice daily with mild abdominal pressure after the operation. There was no unanticipated postoperative mortality in any group of rats.

### 2.3. Urodynamics

Urodynamics in rats were performed as described previously [17]. Briefly, a midline laparotomy was performed to expose the bladder under 1.5% isoflurane anesthesia. A 6-0 surgical suture was applied at the top of the bladder for purse-string closure, a small cystostomy was made in its center, and a polyethylene catheter (PE50) was inserted into the bladder dome. After closing the peritoneal and abdominal wall muscle layers, the PE50 catheter was passed subcutaneously to the interscapular region. The bladder catheter was checked for leakage. After 3 h of recovery in the metabolic cage, the bladder was emptied through the catheter before the experiment and then filled with room-temperature saline solution at a constant filling rate of 100 μL/min. After a 30 min equilibration period, intravesical pressure and urine weight were recorded for 60 min. Subsequently, residual volumes (RVs) were measured during three consecutive voiding cycles by disconnecting the catheter and allowing the bladder contents to drain naturally at equilibrium. Bladder parameters were measured according to the definitions of Andersson et al. [18] and Abrams et al. [19].

### 2.4. Histology

Bladders were harvested at respective time points, fixed in formalin 4%, and embedded in paraffin. Paraffin sections (5 μm) were dewaxed in xylene and rehydrated with ethanol. Slides were subsequently stained with hematoxylin and eosin (HE).

Masson trichrome staining was performed according to the standard protocol. Briefly, after deparaffinization and rehydration, bladder slides of 5 μm were stained with Weigert’s iron hematoxylin for 10 min. After rinsing, the application of the Biebrich scarlet-acid fuchsin solution was carried out for 15 min. Slides were washed, placed in the phosphomolybdic-phosphotungstic acid solution for 15 min, and subsequently placed in the aniline blue solution for 10 min. Five images were randomly taken, and smooth muscle content in the bladder was calculated with Image-Pro Plus (6.0; Media Cybernetics, Silver Spring, MD, USA).

### 2.5. Total RNA Extraction and Sequencing

Total RNA was extracted from the bladder with TRIzol (Invitrogen, Carlsbad, CA, USA) according to the manufacturer’s instructions. Total RNA was quantified and assessed by NanoDrop™ One (ThermoFisher, Waltham, MA, USA) and Qubit™ RNA HS Assay Kit (Life Invitrogen, Carlsbad, CA, USA).

Magnetic beads with Oligo (DT) were used to capture mRNA with a polyA structure. The first strand of cDNA was synthesized in the M-MuLV reverse transcriptase system with fragmented mRNA as a template and a random oligonucleotide as a primer. The RNA strand was degraded by RNaseH, and the second strand of cDNA was synthesized by dNTPs in the DNA polymerase I system. The double-stranded cDNA was purified, and the sequencing connector was added. The 5′ cDNA was connected with the UID adaptor. Finally, PCR products were purified with the AMPure XP system, and library quality was assessed on the Agilent 4200 TapeStation (Santa Clara, CA, USA). After passing the library quality inspection, the different libraries were further sequenced on the Illumina PE150 platform.

### 2.6. Quality Control of Raw Data

Raw data (raw reads) in fastq format were processed with fastp, an ultra-fast FASTQ preprocessor with useful quality. Clean data (clean reads) were obtained after routine quality control, adapter trimming, quality filtering, and per-read quality cutting. The data, after quality control, were further processed to remove duplicate reads using the UID label. All the downstream analyses were based on clean data with high quality after the removal of duplicate reads.

### 2.7. Quantification of Gene Expression Level and Differential Expression Analysis

A feature count was used to count the read numbers mapped to each gene. Then, the FPKM of each gene was calculated based on the length of the gene and the read count mapped to this gene. FPKM, or expected number of fragments per kilobase of transcript sequence per million base pairs sequenced, considers the effect of sequencing depth and gene length on the read count at the same time and is currently the most commonly used method for estimating gene expression levels.

Differential expression analysis of two conditions/groups (three biological replicates per condition) was performed using the DESeq R package (1.18.1). DESeq provides statistical routines for determining differential expression in digital gene expression data using a model based on the negative binomial distribution. The resulting *p*-values were adjusted using the Benjamini and Hochberg approach for controlling the false discovery rate. Genes with |log_2_ (FoldChange)| > 1 and an adjusted *p*-value < 0.05 found by DESeq were assigned as differentially expressed.

### 2.8. Function Analysis of the Differentially Expressed Genes

Gene Ontology (GO) and Kyoto Encyclopedia of Genes and Genomes (KEGG) enrichment analysis and visualization of differentially expressed genes were implemented by the clusterProfiler R package, which is a simple-to-use tool to analyze high-throughput data obtained from transcriptomics or proteomics. Genes with a P_adjusted_ less than 0.05 were considered significantly enriched by differentially expressed genes (DEGs). The identified DEGs were then processed for the Gene Set Enrichment Analysis (GSEA) using the R package clusterProfiler (3.8.0), and the thresholds were set as adjusted *p* value < 0.05 and FDR < 0.25.

### 2.9. Short Time-Series Expression Miner (STEM)

Short time-series expression miner is a software program designed for clustering, comparing, and visualizing gene expression data from short time series [20]. We imported the time-dependent matrix of DEGs into STEM software (v1.3.9) according to the instructions, applied the STEM clustering method, and set the maximum number of clusters to 15 for visualization.

### 2.10. Real-Time Quantitative Polymerase Chain Reaction (qRT-PCR)

Total RNA was extracted from bladder tissue with FastPure^®^ Cell/Tissue Total RNA Isolation Kit V2 (RC112, Vazyme, Nanjing, China) and reverse transcribed to cDNA using Hifair^®^ III Reverse Transcriptase (11111ES92; YEASEN, Shanghai, China). Then, the Hieff^®^ qPCR SYBR Green Master Mix (11202ES03, YEASEN) on QuantStudio 6 Flex (Applied Biosystems, Thermofisher, USA) was used to conduct qRT-PCR, and 2^−ΔΔCt^ was used to calculate relative RNA levels. The primers used in the qRT-PCR assay are demonstrated in Table 1.

### 2.11. Protein Extraction and Western Blot

Bladder tissue was homogenized in RIPA lysate (AR0105; Boster, Wuhan, China) containing protease inhibitor cocktails (HY-K0010; MCE, Princeton, NJ, USA). The solution was centrifuged after ultrasonic treatment. The supernatant was gathered, and the protein concentration was determined by BCA assay (AR0146; Boster, Wuhan, China).

For western blot, to ensure consistency in different experiments, 30 μg of proteins were separated by sodium dodecyl sulfate-polyacrylamide gel electrophoresis and transferred to polyvinylidene difluoride membranes. Membranes were blocked in 5% bovine serum albumin (in Tris-buffered saline-Tween solution) for 1 h and then incubated at 4 °C with primary antibodies overnight against anti-IL-1β (1:100, 16806-1-AP, Proteintech, Wuhan, China), anti-IL-18 (1:100, 10663-1-AP, Proteintech, China), anti-Collagen I (1:100, 14695-1-AP, Proteintech, China), anti-Collagen III (1:100, GB111629, Servicebio, Wuhan, China), and β-actin (1:1000, 20536-1-AP; Proteintech). After the blots were incubated with appropriate horseradish peroxidase-labeled secondary antibodies (1:5000, ab6721, Abcam, Cambridge, UK), immunoreactive proteins were caught by the ChemiDoc MP system (Bio-Rad, Hercules, CA, USA). The relative intensity of bands was calculated with homologous β-actin as a control band by ImageJ (v1.5.1, NIH, Bethesda, MD, USA).

### 2.12. Immunohistochemistry

Bladder sections were prepared as described above. Slides were blocked and incubated with anti-ki-67 (1:100, GB111141, Servicebio, China), anti-IL-6 (1:100, GB11117, Servicebio, China), anti-Collagen I (1:100, 14695-1-AP, Proteintech, China), and anti-Collagen III (1:100, GB111629, Servicebio, China). After the slides were incubated with relevant secondary antibodies, five images were randomly photographed and recorded using a BX53F fluorescence microscope (Olympus, Tokyo, Japan). Image-Pro Plus was employed to calculate the ratio between positive areas and the total area for semi-quantitative analysis.

### 2.13. Statistical Analysis

All analyses were carried out with GraphPad Prism 9.0 (GraphPad Software, San Diego, CA, USA). Urodynamics data were presented as means ± 95% confidence interval (CI), and other data were presented as means ± standard deviations (SDs). A one-way ANOVA was used to compare the group means among the 3 groups. *p* < 0.05 represents a statistical difference.

## 3. Results

### 3.1. Urodynamics of the BPNI Rat Model

**Figure 1:**
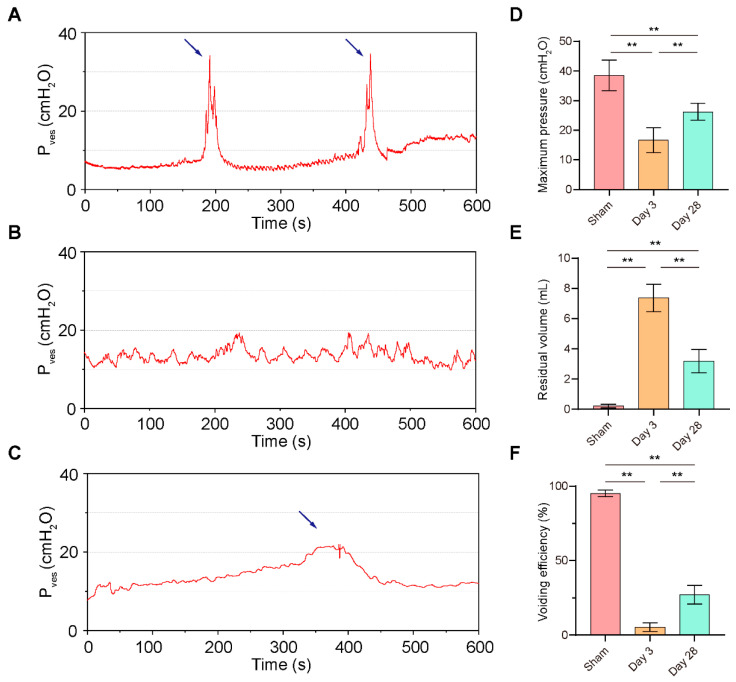
Urodynamics of the BPNI rat model. (**A**–**C**) Representative curves of urodynamics in three groups of rats. (**A**) Sham group; (**B**) 3 days post-BPNI; (**C**) 28 days post-BPNI. The blue arrows showed distinct urination events. Comparison of (**D**) maximum intravesical pressure, (**E**) residual volume, and (**F**) voiding efficiency in three groups of rats. P_ves_ = intravesical pressure; ** *p* < 0.01.

All three groups of rats completed the surgery successfully, and no animals died postoperatively. Urodynamics confirmed a normal postoperative voiding cycle in the sham-operated rats with a maximum intravesical pressure (P_ves max_) of 38.53 cmH_2_O (33.13–43.94) and a voiding efficiency (VE) of 95.20% (92.89–97.51), with almost no residual urine. In contrast, after 3 days of BPNI, a consistent involuntary flow of urine was seen after continuous bladder irrigation; urodynamics showed a high frequency and low amplitude waveform; P_ves max_ decreased to 16.63 cmH_2_O (12.23–21.04); the VE was 5.35% (2.34–8.36); and there was a large amount of residual urine in the bladder, suggesting overflow incontinence. Interestingly, at 4 weeks of BPNI, we observed a partial recovery of voiding function in this group of rats, with a return of P_ves max_ to 26.25 cmH_2_O (23.27–29.23) and a VE of 27.17% (20.54–33.80) (Figure 1). Other cystometric parameters are also summarized in Table 2.

### 3.2. Postoperative Pathological Features of BPNI

**Figure 2:**
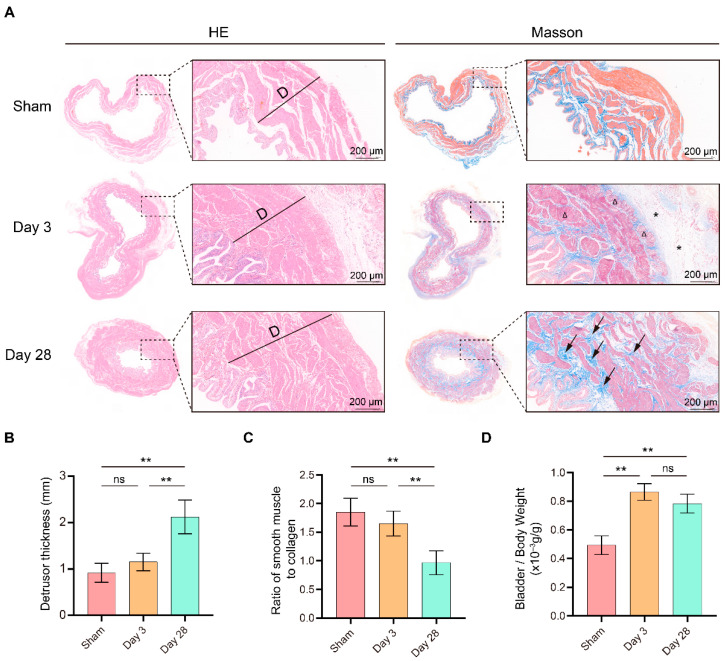
Morphological changes in the bladder after BPNI and sham surgery. (**A**) HE (left) and Masson staining (right) of the BPNI and sham groups. Triangles (Δ) indicate vacuolization of the detrusor, asterisks (*) represent subserosal edema of the bladder, and black arrows represent extensive fibrosis of the bladder wall. Comparison of (**B**) detrusor thickness, (**C**) ratio of smooth muscle to collagen, and (**D**) bladder-to-body weight ratio among the BPNI and sham-operated groups. ** *p* < 0.01, ns = no significance.

Subsequently, we collected bladder tissue from each group of rats for pathological examination (Figure 2A). Compared with sham surgery, a significant thickening of the detrusor and a significantly lower smooth muscle/collagen ratio were detected after 28 days of BPNI (Figure 2B,C). However, no significant changes in detrusor thickness or collagen content were observed 3 days after BPNI. In contrast, there was a significant increase in the bladder-to-body weight ratio at both 3 and 28 days after BPNI (Figure 2D). Therefore, we rechecked the HE and Masson staining in the two BPNI groups to try to explain this difference. Notably, 3 days after BPNI showed mainly subserosal edema and vacuolization of the detrusor, whereas 28 days after BPNI showed mainly extensive fibrosis of the entire bladder wall (Figure 2A).

### 3.3. Comparison of mRNA Expression Patterns in the BPNI and Sham Groups

**Figure 3:**
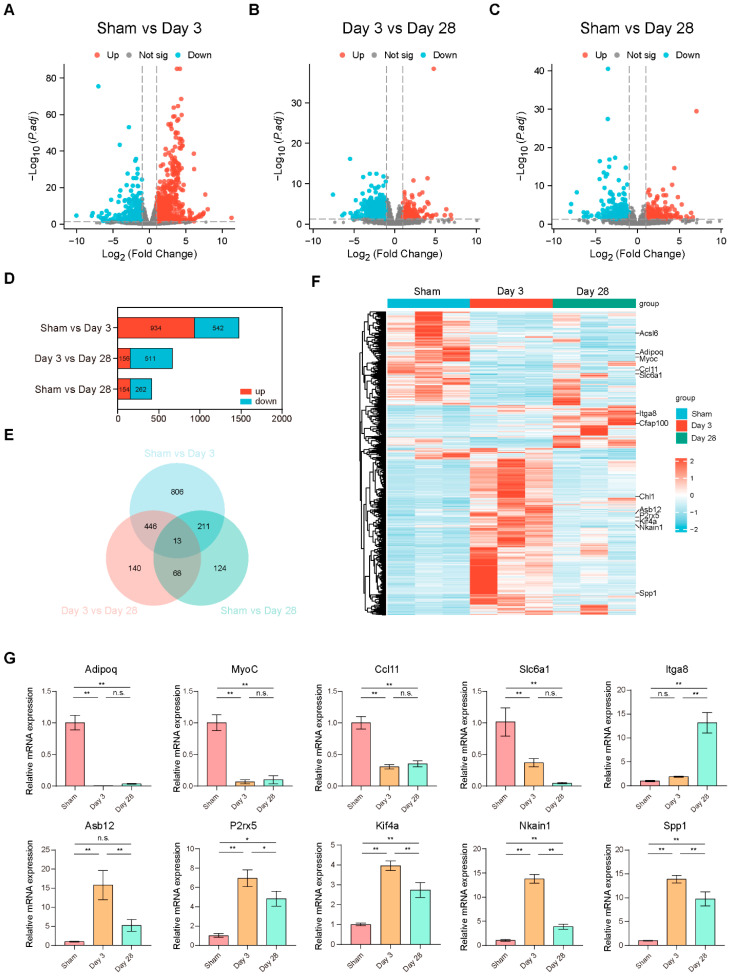
mRNA expression patterns in the BPNI and sham groups. Volcano plots of (**A**) sham group vs. 3 days post-BPNI, (**B**) 3 days post-BPNI vs. 28 days post-BPNI, and (**C**) sham group vs. 28 days post-BPNI. (**D**) Number of upregulated and downregulated DEGs. (**E**) Venn diagrams for pairwise comparison of 3 groups. (**F**) Heat maps showing the characteristic mRNA expression patterns of each group, where the 13 DEGs screened in the Venn diagram are labeled on the right. (**G**) Ten DEGs validated by qRT-PCR. * *p* < 0.05, ** *p* < 0.01, ns = no significance.

To investigate the mechanism of the above urodynamic and morphological alterations occurring at different times after BPNI, we collected bladder tissues from rats in the sham-operated and BPNI groups for RNA-seq. First, we applied volcano plots to visualize the pairwise comparison of the 3 groups and labeled some of the most significant DEGs (Figure 3A–C). A total of 934 genes were upregulated and 542 genes were downregulated in the comparison between the sham group and the 3-day post-BPNI group. Meanwhile, 156 genes were upregulated and 511 genes were downregulated in the comparison between the 3-day and 28-day post-BPNI groups. In the comparison between the sham group and the 28-day post-BPNI group, 154 genes were upregulated and 262 genes were downregulated (Figure 3D). All DEGs are presented in Tables S1–S3.

Subsequently, we used Venn diagrams to take the intersection of the DEGs found in these pairwise comparisons of the 3 groups and found a total of 13 genes that differed in all three comparisons (Figure 3E). The heat map also shows the mRNA expression patterns of different groups, and the 13 DEGs are labeled (Figure 3F). We selected 10 of the mutual 13 DEGs for qRT-PCR validation and found that their expression trends were consistent with RNA-seq, confirming the reliability of the RNA-seq data (Figure 3G). Considering that various types of ion channels and receptors play important roles in bladder sensory function, we summarized a list regarding the review published by Dalghi et al. [21], compared it with our RNA-seq data, and found that ion channels such as Piezo2, Kcnn2, and Trpv2 were differentially expressed among the time points (Table S4).

### 3.4. Functional Analysis of DEGs

**Figure 4:**
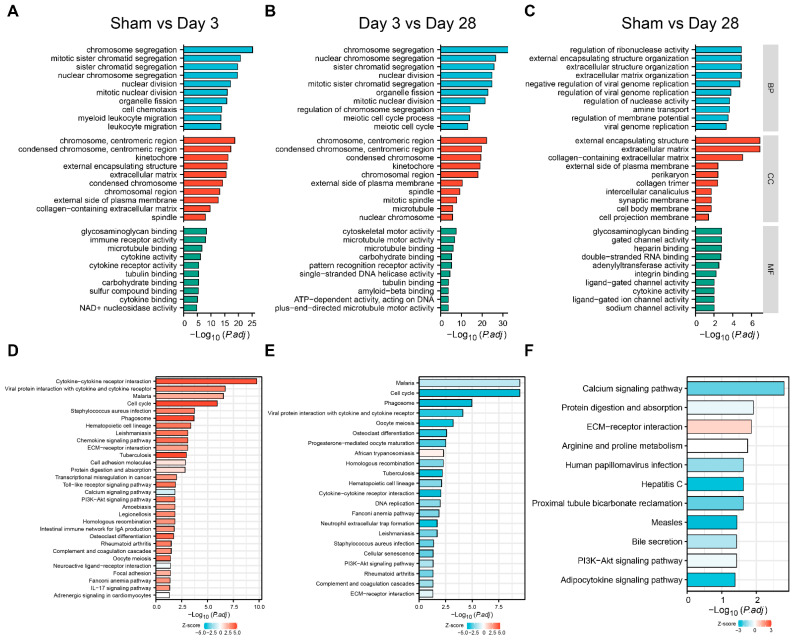
GO and KEGG analyses of DEGs. GO analysis of (**A**) sham group vs. 3 days post-BPNI, (**B**) 3 days post-BPNI vs. 28 days post-BPNI, and (**C**) sham group vs. 28 days post-BPNI. KEGG analysis of (**D**) sham group vs. 3 days post-BPNI, (**E**) 3 days post-BPNI vs. 28 days post-BPNI, and (**F**) sham group vs. 28 days post-BPNI.

To characterize the function of the DEGs in each group, we first included all DEGs in the GO enrichment analysis (Figure 4A–C). A large number of pathways of cellular chromosome segregation and nuclear division, leukocyte migration, and extracellular matrix components were significantly enriched 3 days after BPNI compared to the sham group (Figure 4A). Additionally, at 28 days after BPNI, some cytoskeleton motor activity and microtubule binding pathways were enriched in addition to the above pathways (Figure 4B). Subsequently, we performed KEGG combined with Z-score analysis to clarify the differences in pathway expression (Figure 4D–F). Among the top 30 pathways, except for the calcium signaling pathway, neuroactive ligand-receptor interaction, and adrenergic signaling in cardiomyocytes pathway, which were downregulated, all the pathways represented by the cell cycle, chemokine signaling pathway, Toll-like receptor signaling pathway, PI3K-Akt signaling pathway, and IL-17 signaling pathway were significantly overexpressed (Figure 4D). Interestingly, it appears that, in contrast to 3 days after BPNI, the cell cycle, cytokine-cytokine receptor interaction, DNA replication, and PI3K-Akt signaling pathways were significantly downregulated at 28 days post-BPNI (Figure 4E). Finally, we also compared the pathways differentially expressed in the BPNI postoperative 28-day group and the sham group and found that extracellular matrix organization, collagen trimer, gated channel activity, and other pathways were significantly enriched (Figure 4C). In addition, the KEGG analysis revealed that except for the high expression of the ECM-receptor interaction pathway, the expression of the calcium signaling pathway, protein digestion and absorption, PI3K-Akt signaling pathway, and adipocytokine signaling pathway was inhibited (Figure 4F). All pathways enriched by GO/KEGG are detailed in Tables S5–S7.

**Figure 5:**
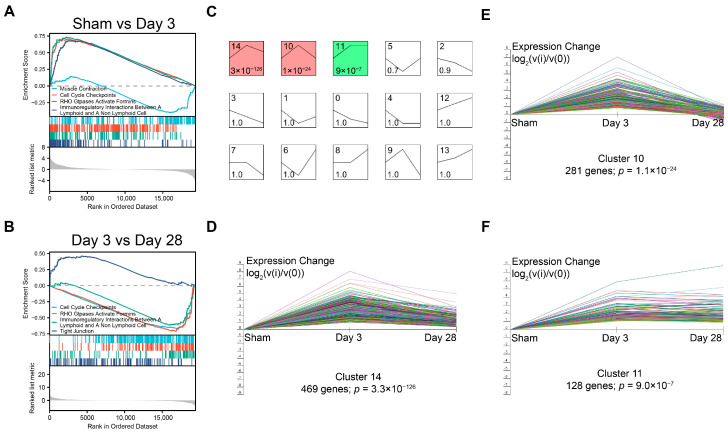
GSEA and the dynamic expression pattern of DEGs. GSEA of (**A**) sham group vs. 3 days post-BPNI and (**B**) 3 days post-BPNI vs. 28 days post-BPNI. (**C**) The short time-series expression miner (STEM) classified all differential expression genes into 15 classes of expression patterns, with (**D**) cluster 14, (**E**) cluster 10, and (**F**) cluster 11 being significant. Each different colored line represents a gene.

Furthermore, we also applied the Gene Set Enrichment Analysis (GSEA) to the DEGs. The muscle contraction dataset was found to be inhibited 3 days after BPNI, while the cell cycle checkpoint, RHO GTPase activation formins, and immunoregulatory interactions pathways were activated (Figure 5A). In contrast, 28 days after BPNI, the above pathways were inhibited and tight junction pathways were activated (Figure 5B). All pathways enriched by GSEA are detailed in Tables S8 and S9.

To analyze the dynamic expression patterns of DEGs at different times after BPNI, we used a short time-series expression miner (STEM) to classify all 1808 DEGs into 15 expression patterns (clusters 0 to 14) (Figure 5C), of which three clusters were significantly different. Cluster 14 contained 469 genes and cluster 10 contained 281 genes, and both expression patterns were similar, with high expression at 3 days after BPNI and expression falling back at 28 days after BPNI. Cluster 11 contained 128 DEGs that were continuously activated after high expression 3 days after BPNI.

### 3.5. Validation of the Main Pathophysiological Processes after BPNI

**Figure 6:**
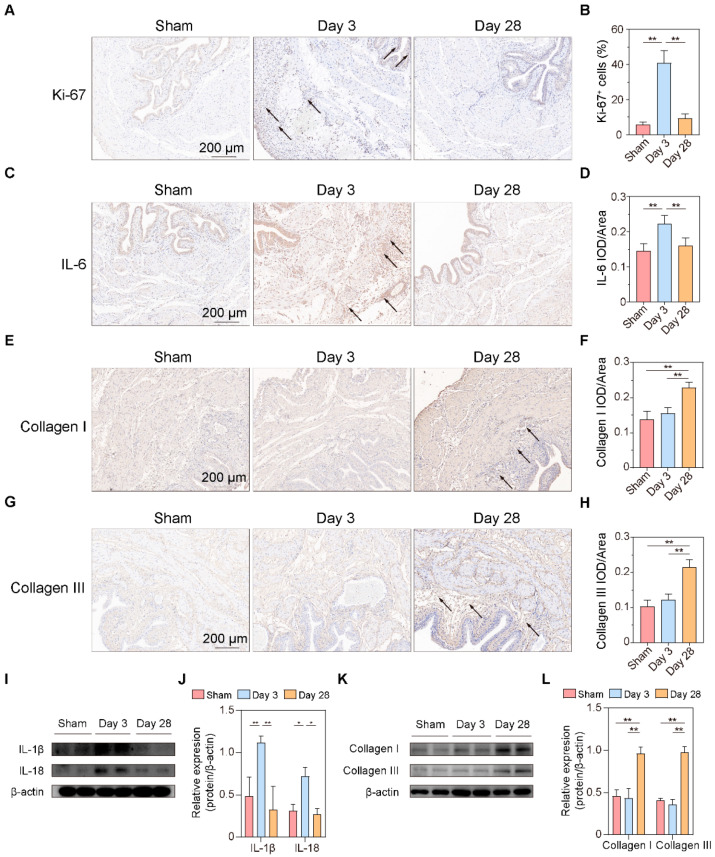
Validation of the main pathophysiological processes after BPNI. (**A**) Immunohistochemical staining of Ki-67 and (**B**) the percentage of Ki-67^+^ cells. (**C**) Immunohistochemical staining and (**D**) integrated optical density (IOD)/area of IL-6. (**E**) Immunohistochemical staining and (**F**) IOD/area of Collagen I. (**G**) Immunohistochemical staining and (**H**) IOD/area of Collagen III. (**I**) Western blot (original images can be found in Figure S1), and (**J**) the relative optical density of IL-1β and IL-18. (**K**) Western blot, and (**L**) the relative optical density of Collagen I and Collagen III. The black arrow shows the positive cells/areas of molecules of interest. * *p* < 0.05, ** *p* < 0.01.

From the above urodynamic, pathological, and bioinformatic analyses, it can be preliminarily concluded that the cell cycle and immune response are in an activated state 3 days after BPNI and that a decrease in muscle contractility begins to occur at this time. Additionally, at 28 days after BPNI, there were a large number of pathways enriched for extracellular structure organization. Therefore, we propose to verify the above pathophysiological processes by immunohistochemistry and western blot in this section (Figure 6). We applied immunohistochemistry to confirm that the cell cycle marker Ki-67 was highly expressed at 3 days after BPNI, while it was at a lower expression level in the sham group and at 28 days after BPNI (Figure 6A,B). Notably, Ki-67-positive cells were present in the basal layers of the urothelium, smooth muscle layer, and subserosal layer. The inflammatory response is also the main pathophysiological process we identified. Immunohistochemistry confirmed high expression of IL-6 3 days after BPNI (Figure 6C,D), while western blot confirmed that IL-1β and IL-18 were also highly expressed at this time (Figure 6I,J). These inflammatory factors returned to normal levels 28 days after BPNI. We also applied immunohistochemistry (Figure 6E–H) and western blot (Figure 6K,L) to determine the alterations of Collagen I and Collagen III in the bladders of the three groups of rats, and the trends were consistent with those demonstrated by Masson staining.

## 4. Discussion

In the present study, we constructed a BPNI rat model and performed urodynamic tests, histological observations, and RNA-seq at 3 and 28 days postoperatively. We found that three days after BPNI, the urodynamics of the rats showed typical features of overflow incontinence, as evidenced by a decrease in P_ves max_, an increase in RV, and a decrease in VE. At the histological level, we observed subserosal edema and vacuolization of the detrusor. At this time, GO/KEGG enrichment analysis confirmed the upregulation of pathways such as cell cycle, chemokine signaling pathway, Toll-like receptor signaling pathway, PI3K-Akt signaling pathway, and IL-17 signaling pathway, while calcium signaling pathway, neuroactive ligand-receptor interaction, and adrenergic signaling in cardiomyocytes pathway were downregulated. At 28 days after BPNI, we observed partial restoration of the voiding pattern in the urodynamics of the rat, as evidenced by the partial rescue of P_ves max_ and VE, as well as a reduction in RV. Correspondingly, the pathology showed significant thickening of the smooth muscle layer of the bladder wall, which was accompanied by a significant increase in collagen deposition. Enrichment analysis showed significant downregulation of the cell cycle, cytokine-cytokine receptor interaction, DNA replication, and PI3K-Akt signaling pathways. Comparing the DEGs in the postoperative 28 days with the sham-operated group, extracellular structure organization was observed, and the above changes were also confirmed by immunohistochemistry. Overall, we mapped the transcriptomic landscape at 3 and 28 days after bladder denervation, and the associated findings provide useful insights into the development of therapeutic targets for DU.

The micturition process depends on the synergistic work of the detrusor and sphincter and is mediated by a complex system of neural control located in the peripheral ganglia, the spinal cord, and the brain [22]. Intravesical signals are transmitted through the urinary epithelium to the afferent nerves and subsequently from the spinal cord to the brain and ultimately to the pelvic nerves (parasympathetic), the hypogastric nerves (sympathetic), and the pubic nerves (somatic) to control the detrusor and sphincter working in tandem. In the pelvic nerve, there is a mixture of afferent nerves and parasympathetic neurons that innervate the contraction of the detrusor [16]. Therefore, in theory, the BPNI model can damage both afferent and efferent nerves, resulting in the inability to transmit information in the bladder to the micturition center while the detrusor is denervated. In this scenario, the bladder of rats after BPNI is significantly overfilled and unable to complete the voiding process autonomously. Urodynamics is the main method to evaluate lower urinary tract dysfunction [18]. In DU, in particular, it is mainly characterized by a decrease in VE and contractility of the detrusor muscle, with or without a decrease in urinary flow rate. In the present study, we followed a previously reported protocol for the construction of a BPNI rat model and selected two time points, 3 and 28 days postoperatively, for urodynamic assessment in the awake state. It is worth noting that previous studies have mostly selected seven days postoperatively as the acute period after BPNI injury. Considering that early intervention may have a higher clinical value, we first assessed urodynamic changes three days after BPNI. It was found that the urodynamic performance at three days after BPNI was essentially the same as that reported in previous studies at seven days after surgery, with overflow incontinence as well as a significant decrease in VE, suggesting that pathophysiological changes occurring after bladder denervation may happen earlier. Dewulf et al. demonstrated that there was no further recovery of voiding function in rats at nine weeks after BPNI compared with three weeks after surgery [16]. Payne et al. similarly demonstrated that there was no difference in the degree of recovery of sensory and motor axons in the pelvis at 4 and 12 weeks of injury [23]. Therefore, we selected 28 days (four weeks) after BPNI as a representative time point of the chronic phase for our study. Consistent with previous reports, our study found partial recovery of all major urodynamic parameters at 28 days after BPNI, but it was still far from the sham-operated group, which may be related to the difficulty of recovery after nerve injury [15]. Notably, the existing animal models of DU almost always apply the method of increasing abdominal pressure without using intermittent catheterization to drain urine, probably due to the researchers’ concern that repeated anesthesia would affect the original urinary pattern of the animals. Nonetheless, several previous studies have demonstrated that the method of increasing abdominal pressure is potentially associated with complications such as upper urinary tract injury, hydronephrosis, urinary tract infection, and stones [24,25]. Therefore, intermittent catheterization, as a safer and more reliable method, may have a different molecular expression pattern in the bladder wall than the method of increasing abdominal pressure, which deserves to be further explored and clarified in future studies.

Furthermore, we evaluated the postoperative pathological changes. Subserosal edema and vacuolization of smooth muscle cells were the main pathological features in the early post-denervation period [16]. In addition, we found a thickening of the bladder wall and an increase in the bladder weight ratio in BPNI, consistent with previous reports, suggesting a possible compensatory effect of the detrusor muscle [16]. An interesting experiment was performed by Stover et al., who demonstrated PI3K/Akt pathway-dependent hyperplasia in rat bladder smooth muscle cells exposed to continuous in vitro cyclic stress (40 cmH_2_O) [26]. This is consistent with Anderson et al., who reported that hypertrophy of the detrusor muscle is not directly related to parasympathetic nerve nutrition or autonomic contraction of smooth muscle and that bladder wall stress may be the main cause of smooth muscle hypertrophy [27]. This suggests that overfilling of the bladder due to BPNI may be the mechanism of detrusor hyperplasia. As DU continued to progress, the proliferation of mesenchymal cells and massive secretion of collagen in the submucosa were observed 28 days after BPNI, accompanied by a loss of vacuolization of smooth muscle cells, manifesting as fibrosis of the bladder wall. Immunohistochemistry also confirmed the high expression of Collagen I and Collagen III in the submucosa. The above pathological manifestations were similar to those reported by Dewulf et al. [16].

The urothelium acts as an effector, sensing environmental changes such as pressure, temperature, pH, and chemical changes in the bladder through various types of receptors and ion channels expressed on its superficial layer [28]. Subsequently, the basal urothelium can release neurotransmitters or small molecules such as ATP, NO, Ach, and NGF to transmit bladder-filling information to afferent nerves [21]. In recent years, researchers have also discovered that the urothelium can directly interact with interstitial cells in the submucosa and smooth muscle further out, which makes the urothelium form a veritable “crosstalk network” with various types of cells. Here, we have compiled a list of important receptors and channels in the urothelium and surrounding various types of cells based on a comprehensive review by Dalghi et al. [21]. We compared the list with our RNA-seq data and found differences in the expression of channels or receptors such as Piezo2, Kcnn2, and Trpv2. This suggested that in addition to the channels that have been reported to be involved in bladder sensation (e.g., TRPV1, TPRV4, etc.), these non-classical channels or receptors may also play a role in the onset and progression of DU.

Subsequently, we performed GO/KEGG enrichment analysis of the screened DEGs and found that most signaling pathways were activated three days after BPNI. Among them, cell cycle-related pathways were significantly overexpressed, which was also confirmed by GSEA and immunohistochemistry for Ki-67. Kullmann et al. reported a large number of Ki-67-expressing cells in the basal layer of the urothelium in the bladder within 1–3 days of spinal cord injury, but they almost completely disappeared 28 days after SCI, reflecting the rapid repair process of the urothelium after the loss of innervation [29]. This suggests that the urothelium after bladder denervation appears to be in an active proliferative state, regardless of the location of the nerve injury. Many Ki-67^+^ cells were also observed in the subserosa layer, and in combination with HE staining and immunohistochemistry for IL-6, this group of cells was considered to be proliferatively active immune cells. In addition, leukocyte migration, immune receptor activity, cytokine-cytokine receptor interaction, and Toll-like receptor signaling pathways were significantly enriched three days after BPNI, which may be related to the inflammatory response that occurs in the acute phase after bladder denervation. Li et al. reported a rat model of sacral nerve injury that showed a significant increase in bladder volume and residual urine at one and four weeks after modeling [30]. PCR results showed that one week after injury, the expression of pro-inflammatory factors IL-1β, IL-6, and TNF-a in the bladder was significantly elevated. Metcalfe et al. observed an increase in the number of cells in the bladder wall in a model of bladder outlet obstruction, suggesting that the inflammatory response may play a more important role in the early stages of the disease [31]. At 28 days post-BPNI, a “new homeostasis” appears to have returned within the bladder tissue, with downregulation of almost all pathways, including cell cycle, immune response, cytokine-cytokine receptor interaction, and other signaling pathways. However, extracellular matrix organization, a large number of gated channel activities, and calcium signaling pathways were abnormal compared to the sham-operated group. Contraction of the detrusor is dependent on increased intracellular calcium ions and the activation of calcium signaling pathways [32]. Given that pathology at 28 days after BPNI confirmed extensive fibrosis in bladder wall tissue, we can speculate that fibrosis may affect bladder perception through gated channel activity and bladder contractile function through calcium signaling pathways. STEM analysis is commonly used for differential genetic time-dependent changes. For example, Liu et al. applied STEM analysis to determine gene expression patterns in the left atrium of db/db mice at 12, 14, and 16 weeks [33]. We found that most DEGs were in an ascending followed by a descending expression pattern, which is generally consistent with that found by GO/KEGG analysis and GSEA.

In general, our data demonstrate that BPNI causes DU with a possible “n” pattern of gene expression, which is generally consistent with the dynamic changes found in urodynamics and pathology. The proliferation and inflammatory response of various types of cells in the bladder wall may be important targets for intervention in the early stages of DU development. At the same time, the prevention of increased pressure in the bladder causing detrusor hypertrophy by drainage of urine through clean intermittent catheterization may also be one of the most reliable therapeutic tools. In the chronic phase of DU, the focus needs to be on fibrosis progression, disruption of ion channels and receptors within the bladder wall, and downregulation of calcium signaling pathways. However, we still believe that early intervention is essential, as the reversal of fibrosis is an equally challenging problem.

There are still some limitations to the present study. First, the comparison regarding voiding-associated ion channels and receptors is based on differential genetic screening criteria and is preliminary and cursory. Many receptors and channels are not unique to a particular cell type, but we believe that this comparison at least reflects the trend of variation in some classes of channels in the bladder. Second, our study found that immune responses and extracellular matrix remodeling within the bladder wall are the main alterations in the early and chronic stages of DU. However, these results are mainly based on RNA-seq, a “black box” approach that makes it difficult to clarify the role of a specific cell population in the development of DU. In the future, single-cell RNA-sequencing of conditional knockout mice may play an important role in studying the function of a receptor or ion channel in a particular cell. Additionally, analysis of immune cell interactions with uroepithelial cells, fibroblasts, and smooth muscle cells, the study of specific ligand-receptor pairs through cellular communication, and in vitro/in vivo drug screening and validation may be optional experimental schemes for identifying molecules critical for DU onset and progression. Third, the present study focused more on describing changes in phenotype, gene expression, and pathways during the development of DU, but how these changes occur remains to be further investigated.

## 5. Conclusions

We first mapped the transcriptome of BPNI leading to neurogenic DU in the early and chronic stages, confirming that immune response and extracellular matrix remodeling occurring within the bladder wall are the main alterations in the early and chronic stages of DU, respectively. The related results have important clinical implications for identifying therapeutic targets and advocating early intervention in DU.

## Figures and Tables

**Table 1:** Primer information.

Gene	Primer Sequence (5′ to 3′)	Number of Bases
Adipoq	Forward: CCACCCAAGGAAACTTGTGC	20
	Reverse: GACCAAGAACACCTGCGTCT	20
MyoC	Forward: AAAGAGGGAGACAAAGGATGTGG	23
	Reverse: CCTGTGCCAACCGTGTCAAT	20
Ccl11	Forward: TGGCTCACCCAGGTTCCATC	20
	Reverse: TGCTTTGTGGCATCCTGGAC	20
Slc6a1	Forward: GCCAGTGACAAGCCCAAAAC	20
	Reverse: GGAATTAGGAAGGCCCCACC	20
Itga8	Forward: TACAACGGAAACGCCAGAGG	20
	Reverse: CCCCGACAAGTAAATCTGGGT	21
Asb12	Forward: GAGCGAACATCTACCTCCCA	20
	Reverse: AGTGACCGTGGAGTAGCTCG	20
P2rx5	Forward: ATCGTGACTCCTAACCAGCG	20
	Reverse: CAGTTTTCAGTCCGTGCCCA	20
Kif4a	Forward: AAGGGACGTTTAGTTTGTATTGG	23
	Reverse: TAGGAGACTCGGAACTCGCT	20
Nkain1	Forward: AGCCTCTGTACACGTCTGGG	20
	Reverse: TCATCGTGATCCAAGCCTCC	20
Spp1	Forward: CCAGCCAAGGACCAACTACA	20
	Reverse: AGTGTTTGCTGTAATGCGCC	20
β-actin	Forward: ATCATTGCTCCTCCTGAGCG	20
	Reverse: GAAAGGGTGTAAAACGCAGCTC	22

**Table 2:** Analyses of cystometry.

Parameters, Mean (95% CI)	Sham	BPNI 3 Days	BPNI 28 Days	*p*
Maximum intravesical pressure, cmH_2_O	38.53 (33.13–43.94)	16.63 (12.23–21.04) **	26.25 (23.27–29.23) **^, ##^	<0.001
Basal pressure, cmH_2_O	8.55 (6.41–10.69)	9.73 (7.67–11.80)	9.53 (7.70–11.36)	0.611
Intercontractile interval, s	199.7 (176.3–223.1)	n.a.	338.3 (317.8–358.9) **	0.002
Micturition volume, g	0.82 (0.55–1.09)	n.a.	0.96 (0.77–1.14)	0.288
Residual volume, mL	0.22 (0.09–0.34)	7.37 (6.42–8.32) **	3.18 (2.38–4.00) **^, ##^	<0.001
Voiding efficiency, %	95.20 (92.89–97.51)	5.35 (2.34–8.36) **	27.17 (20.54–33.80) **^, ##^	<0.001

Values are represented as the mean (Lower 95% CI—Upper 95% CI) with *n* = 6 per group. ** *p* < 0.01 compared to the sham group; ## *p* < 0.01 compared to the BPNI at 3 days.

## Data Availability

Data in this study can be obtained by request and with permission from the corresponding author.

## Supplementary Materials

The following supporting information can be downloaded at: https://www.mdpi.com/article/10.3390/biom13081260/s1. Table S1: DEGs of sham group vs. 3 days post-BPNI; Table S2: DEGs of 3 days post-BPNI vs. 28 days post-BPNI; Table S3: DEGs of sham group vs. 28 days post-BPNI; Table S4: Partial channels and receptors of urothelium; Table S5: GO/KEGG analysis of sham group vs. 3 days post-BPNI; Table S6: GO/KEGG analysis of 3 days post-BPNI vs. 28 days post-BPNI; Table S7: GO/KEGG analysis of sham group vs. 28 days post-BPNI; Table S8: GSEA of sham group vs. 3 days post-BPNI; and Table S9: GSEA of 3 days post-BPNI vs. 28 days post-BPNI; Figure S1: Original western blots for Figure 6I,K.
