# Supplementary material for: Transcriptomics Reveals Molecular Features of the Bilateral Pelvic Nerve Injury Rat Model of Detrusor Underactivity
Source: Biomolecules. 2023 Aug 18;13(8):1260. doi: 10.3390/biom13081260 (PMC10452637; doi:10.3390/biom13081260)

WB raw images with labels

Figure 6i

1. Chemiluminescence, colorimetric and merge images of IL-1β (32kDa)

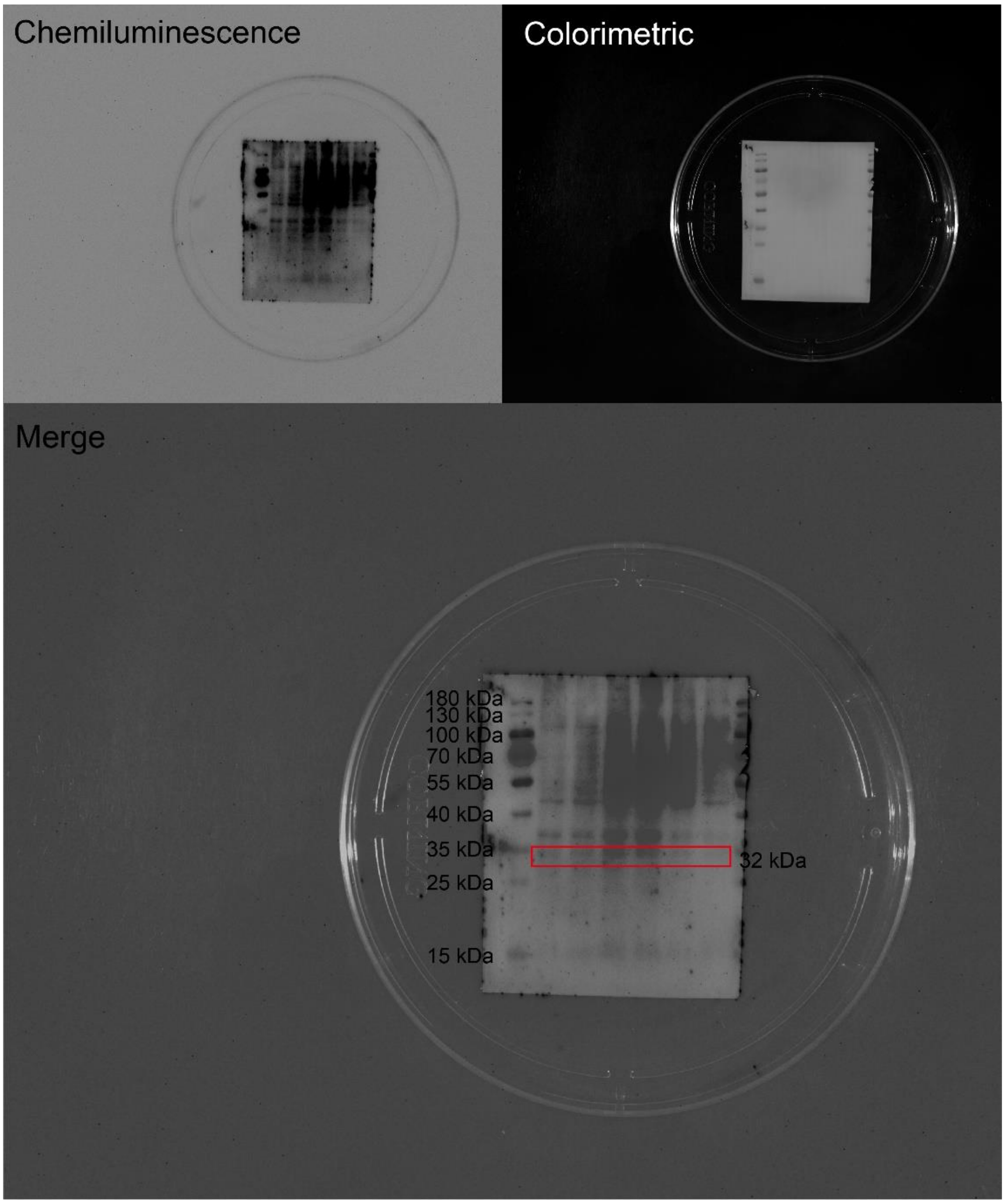

2. Chemiluminescence, colorimetric and merge images of IL-18 (22kDa)

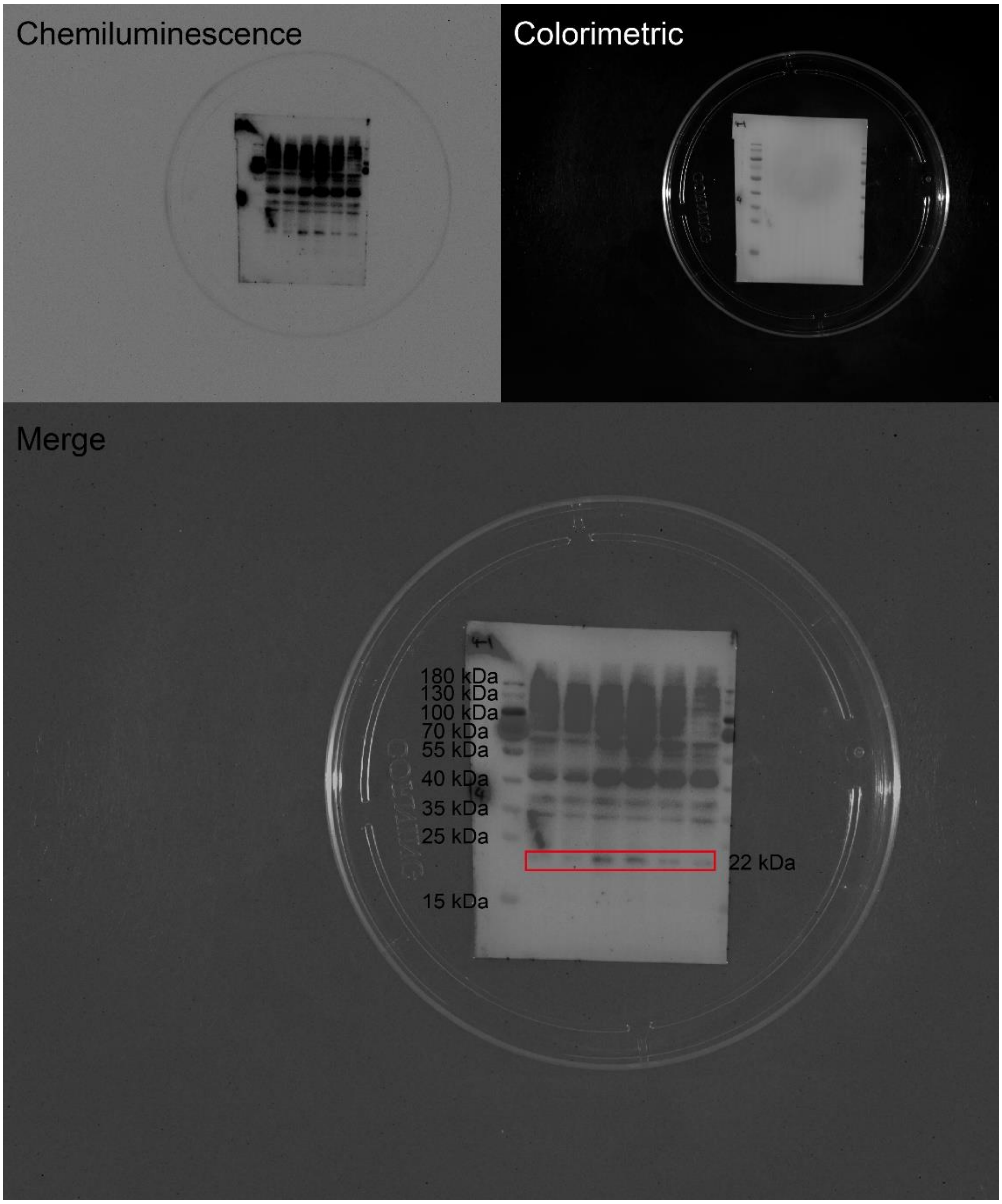

3. Chemiluminescence, colorimetric and merge images of β-actin (45kDa)

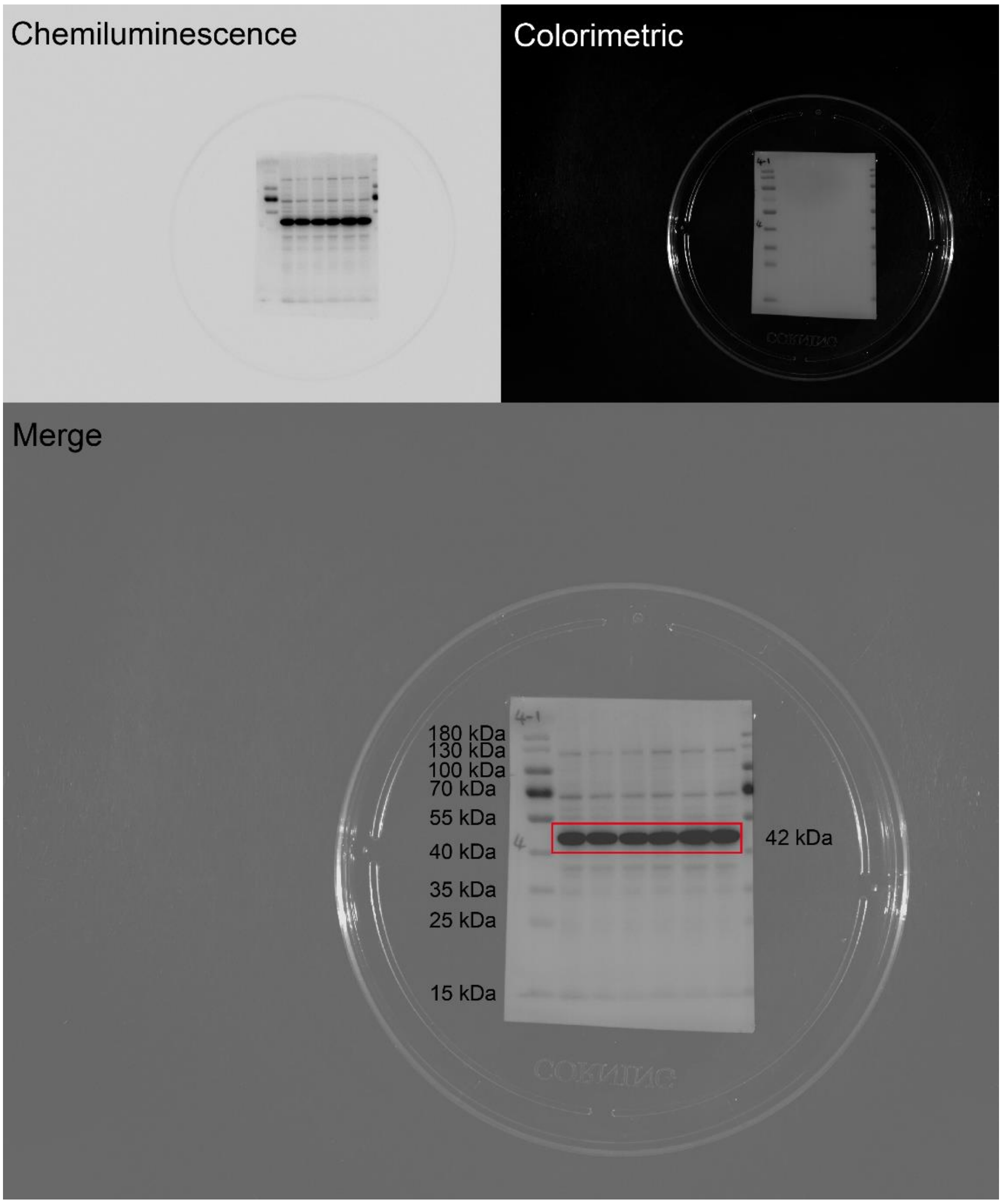

Figure 6k

1. Chemiluminescence, colorimetric and merge images of collagen I (120 kDa)

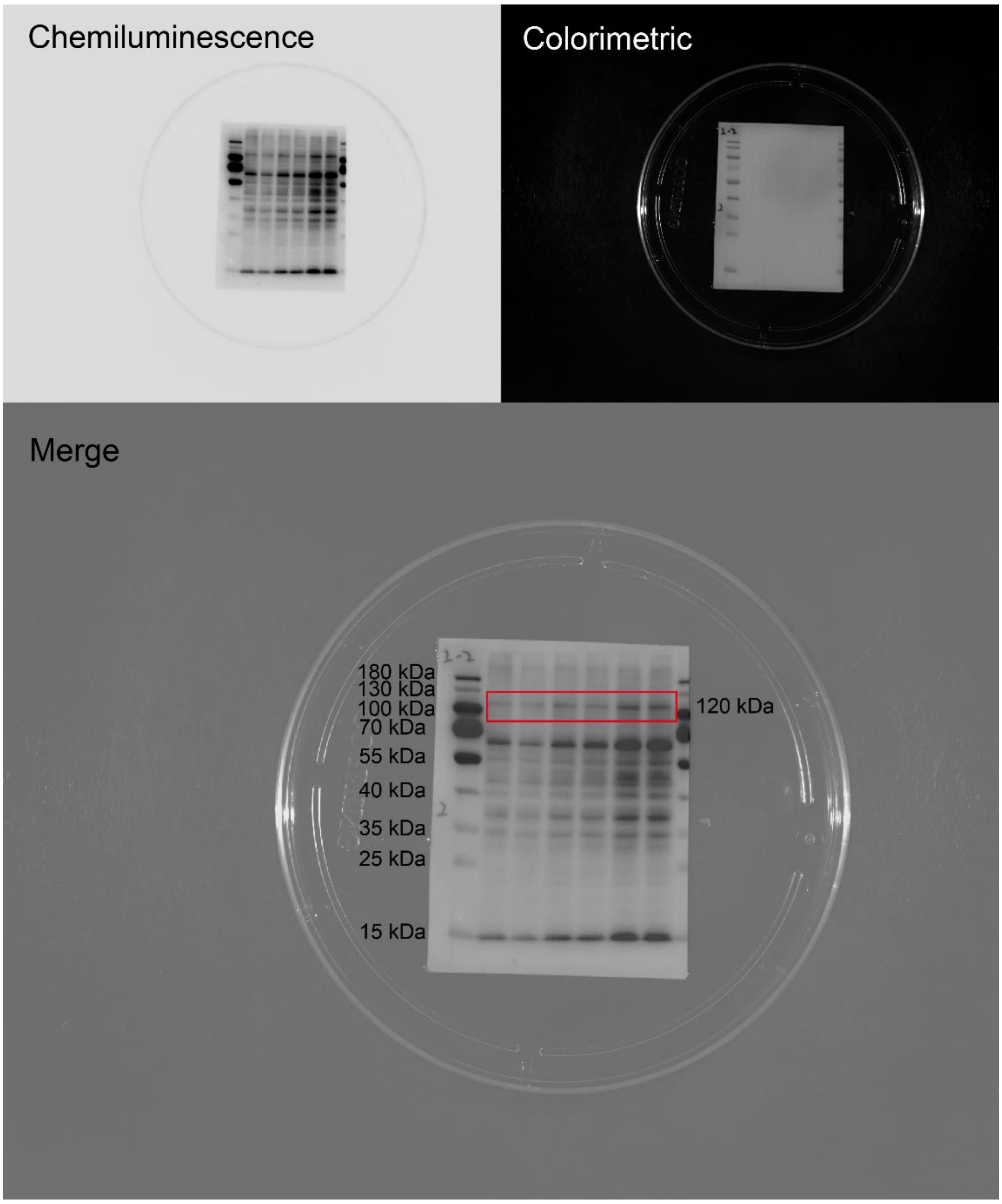

2. Chemiluminescence, colorimetric and merge images of collagen III (138kDa)

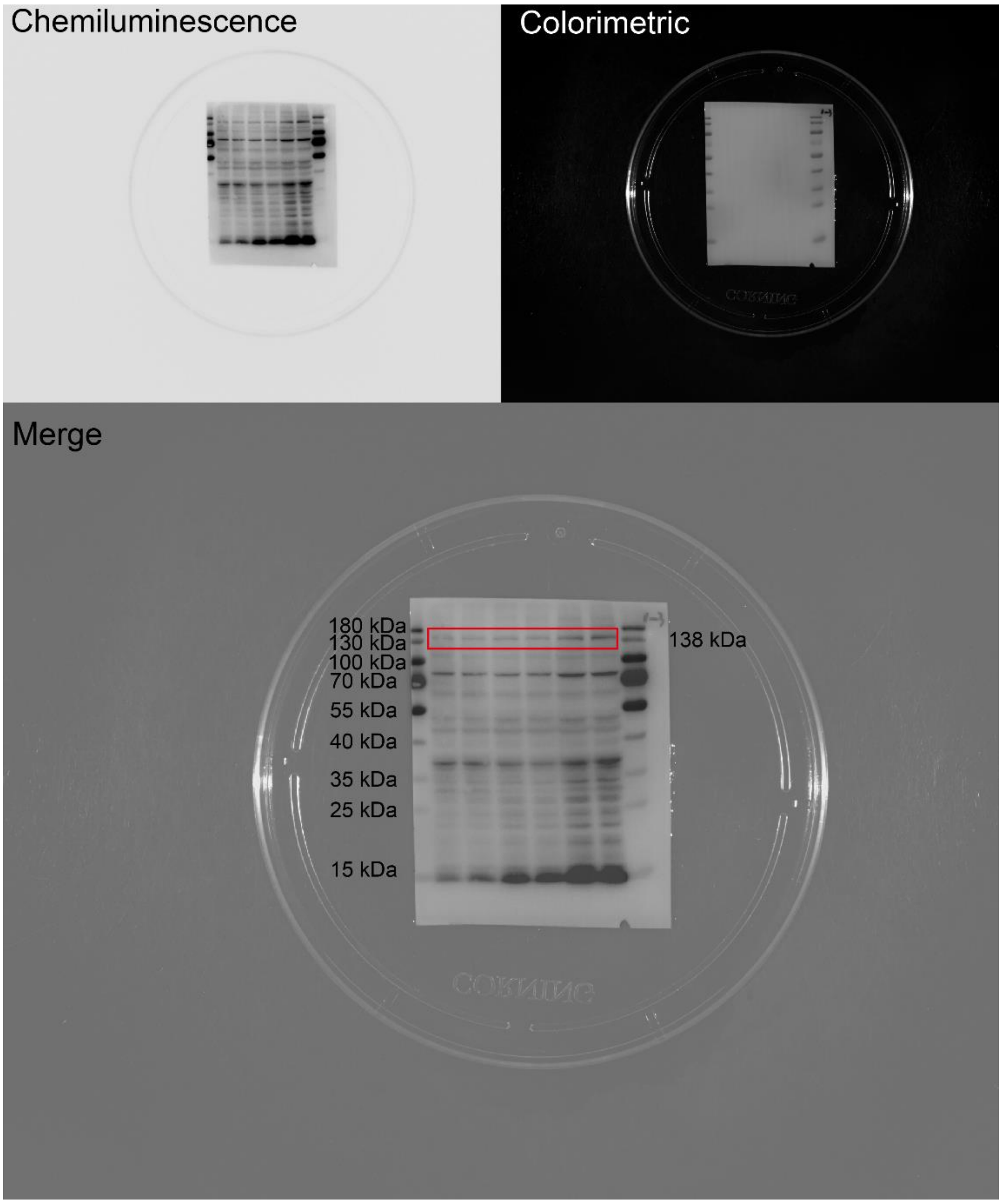

3. Chemiluminescence, colorimetric and merge images of β-actin (45kDa)

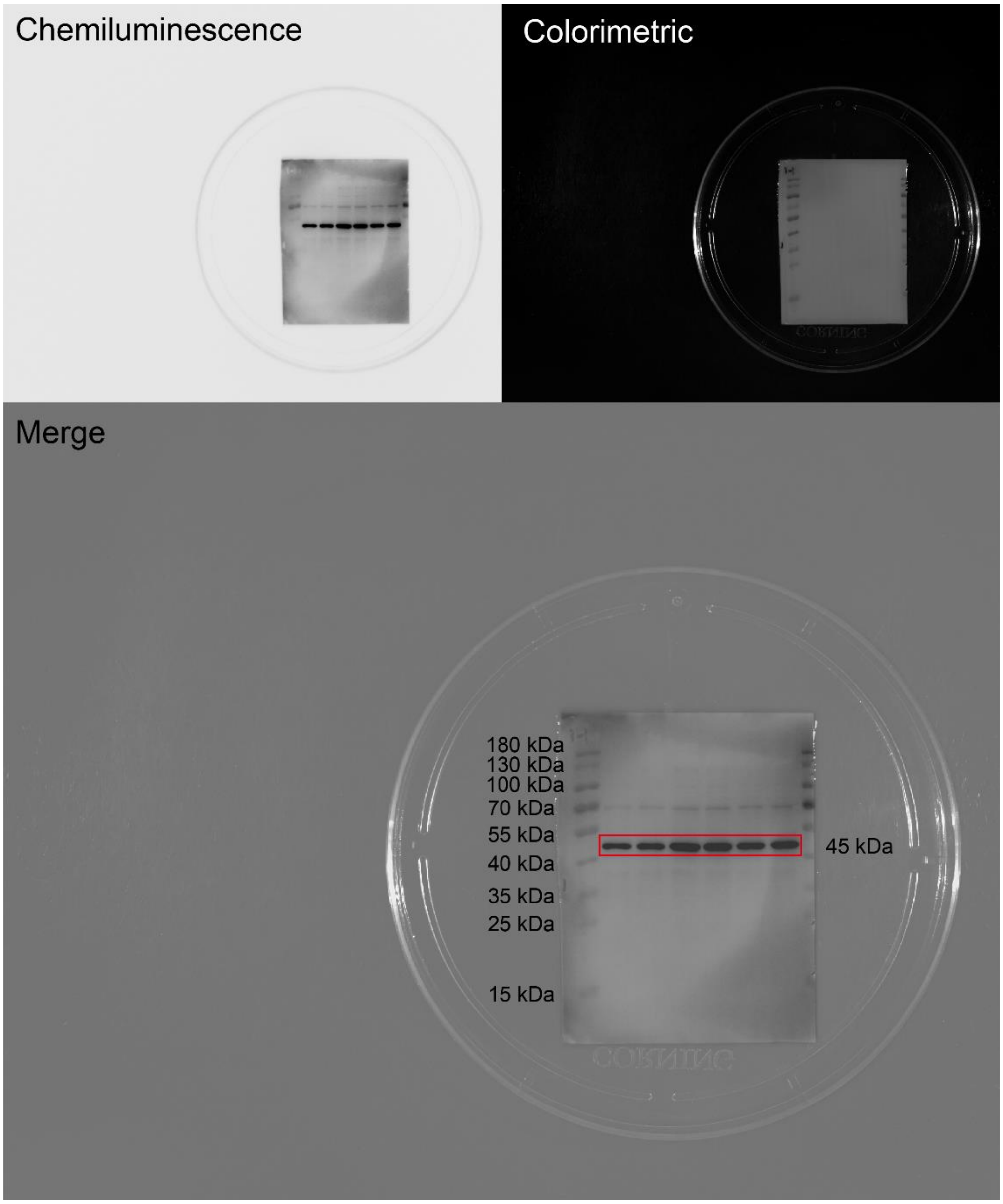

Supplement: Supplementary file 1 [file biomolecules-13-01260-s001.zip › Figure S1 Original western blots for Figure 6I,K.pdf]
